# Effect of an Oral Health Programme on Oral Health, Oral Intake, and Nutrition in Patients with Stroke and Dysphagia in Taiwan: A Randomised Controlled Trial

**DOI:** 10.3390/ijerph16122228

**Published:** 2019-06-24

**Authors:** Hsiao-Jung Chen, Jean-Lon Chen, Chung-Yao Chen, Megan Lee, Wei-Han Chang, Tzu-Ting Huang

**Affiliations:** 1 Graduate Institute of Clinical Medical Sciences, Nursing, Chang Gung University, Taoyuan City 33378 Taiwan; crchen51@gmail.com; 2 Department of Physical Medicine & Rehabilitation, Taoyuan Chang Gung Memorial Hospital, Taoyuan City 33378, Taiwan; bigmac1479@gmail.com (J.-L.C.); m7252@adm.cgmh.org.tw (W.-H.C.); 3 Department of Medicine, College of Medicine, Chang Gung University, Taoyuan City 33378, Taiwan; 4 Department of Physical Medicine & Rehabilitation, Chang Gung Memorial Hospital, Keelung, Keelung City 20401, Taiwan; jongyau@cgmh.org.tw; 5 Department of Biochemistry, University of Washington, Seattle, WA 98134, USA; meganlee328@gmail.com; 6 School of Nursing, Healthy Aging Research Center School of Nursing, College of Medicine, Chang Gung University, Taoyuan City 33378, Taiwan; 7 Department of Dementia Center, Chang Gung Memorial Hospital Linkou Medical Center, Taoyuan City 33378, Taiwan

**Keywords:** oral health, oral intake, stroke, dysphagia, randomised controlled trial

## Abstract

No previous studies have evaluated an oral health programme, before swallowing therapy, in patients with stroke and dysphagia in Taiwan. This randomised controlled trial evaluated the effect of an oral health programme (i.e., sputum assessment, Bass method-based tooth brushing, and tooth coating with fluoride toothpaste) before swallowing therapy. Sixty-six patients with stroke (23 female, 43 male) in our rehabilitation ward, who underwent nasogastric tube insertion, were assigned randomly to an oral care group (*n* = 33) and a control group (*n* = 33). Demographic data, oral health assessment, Functional Oral Intake Scale (FOIS) scores, Mini-Nutritional Assessment-Short Form (MNA-SF) scores, and nasogastric tube removal rates were compared between groups. We evaluated outcomes using generalised estimating equation analysis. Three weeks post-implementation, the oral care group had significant oral health improvements relative to the control group (95% CI =−2.69 to −1.25, Wald **χ^2^** = 29.02, *p* < 0.001). There was no difference in the FOIS (95% CI = −0.16 to 0.89, Wald **χ^2^** = 1.86, *p* > 0.05), MNA-SF (95% CI =−0.35 to 0.53, Wald **χ^2^** =−0.17, *p* > 0.05), and nasogastric tube removal (*p* > 0.05) between groups. The oral care group had a higher, but non-significant FOIS score (3.94 vs 3.52) (*p* > 0.05). Routine oral health programmes implemented during stroke rehabilitation in patients with dysphagia may promote oral health and maintain oral intake.

## 1. Introduction

Chewing and swallowing disorders commonly develop after a stroke. Concurrently, health problems associated with dysphagia increase with age. A stroke can occur in the brain stem or other brain areas and can be bilateral or unilateral. Previous studies have shown that the severity of dysphagia is related to the location of the stroke in the brain [1,2]. Further, dysphagia, advanced age, and restricted upper limb movement are all important risk factors for malnutrition [3].

In the United States, 10%–30% of all patients with acute stroke are treated with indwelling nasogastric tubes [4,5]. In Taiwan, approximately 31% of all patients with acute stroke are treated with indwelling nasogastric tubes [6]. Patients and caregivers tend to ignore the oral health of patients requiring tube feeding and, therefore, ignore the need for meticulous oral healthcare [7]. Therefore, patients with indwelling nasogastric tubes often experience oral health-related problems, such as reduced salivary flow, dry mouth, and dental caries [8]. Moreover, the fibre arrangement and physiology of the human masticatory muscles are related to diet and eating [9], and the effects of temporomandibular joint arthritis have been shown to lead to higher rates of functional disability [10]. Previous studies have shown that the refusal of dental treatment because of carious lesions may lead to decreased oral health-related quality of life (OHRQoL) due to the resulting increase in pain and halitosis, decrease in chewing performance, and poor sense of cleanliness [11,12]. These factors influence the swallowing function and oral food intake in affected patients. As a result, patients with stroke must often rely on nasogastric tube-based nutrition for an extended period.

Good oral healthcare is known to also improve food intake [13,14,15,16]. For example, oral care can improve oral sensation, relieve dry mouth, and increase salivary flow, thereby promoting appetite [9]. Mechanical stimulation of the oral cavity can also have an indirect training effect on the recovery of food intake and swallowing [17].

In addition, oral care can have a significant effect on swallowing, food intake, and prevention of malnutrition [17]. Malnutrition is a common problem occurring after acute stroke, with an incidence of 8%–34% in the United States [18] and approximately 51% in Taiwan [6]. Therefore, the implementation of an oral care programme is very important for patients with stroke who have indwelling nasogastric tubes.

According to the literature, oral health programmes are typically designed for vulnerable populations, such as patients with psychiatric disorders [19,20], those with cancer and chronic diseases [21,22], those susceptible to aspiration pneumonia [23,24], and those requiring long-term care [25]. A randomised controlled trial (RCT) concluded that relatively few studies on oral health programmes have been conducted to date [19,20,21,22,23,24,25]. In particular, there is a lack of care programmes specifically designed for patients who have stroke with dysphagia. At present, the results of two studies regarding the effect of oral health promotion in patients with stroke showed a significant improvement in oral hygiene and oral food intake [14,15]. From these reports, it can be concluded that it is important to maintain oral hygiene and a good oral state to maintain good tactile sensation and taste during food intake [13,16]. Further, reducing halitosis can increase oral comfort, increase the willingness to eat, and finally contribute to the removal of nasogastric tubes, thus resulting in improved nutritional status.

By integrating the currently available literature, we have created the following oral care programme to be followed prior to swallowing therapy: 1. Preparation of the suction machine and tube, followed by assessment of the sputum of the oral cavity. The sputum can be suctioned, to allow better visualisation [26]; 2. selection of the appropriate oral cleaning tool (dental floss and/or interdental brush) [15]; 3. Brushing teeth using the Bass method [27] and the use of a suction tube to remove saliva. This study aimed at examining the effectiveness of this oral health programme by assessing specific outcome variables (oral health, functional oral intake, nutritional status, and rate of nasogastric tube removal) among patients who have stroke with dysphagia. The hypotheses of the present RCT are: H0 that there is a similar effect on oral health, functional oral intake, nutritional status, and percentage of nasogastric tube removals between the oral health programme and the use of an instructional manual to promote eating (i.e., oral health programme is ineffective); and H1: that there is greater oral health, functional oral intake, nutritional status, and more nasogastric tube removals in those receiving a 3 week oral health programme compared to those who receive an instructional manual only.

## 2. Materials and Methods

### 2.1. Sample Size Calculation

G Power analysis was performed to calculate the minimum sample size. Sixty-five participants were sufficient to statistically identify a difference of 2.7% in the prevalence of the severe Functional Oral Intake Scale (FOIS; levels 4–5 and levels 6–7) between the control and intervention groups, with a statistical power of 80% and a 5% level of significance. The effect size was set at 0.3–0.4 [28]. In addition, a 5% drop-out rate and an intra-ward correlation of 0.5 based on an intervention conducted in Taiwan in two groups at three time points were taken into account [29]. The 2.7% prevalence was selected, as it was the smallest difference (control group, 31.8%; intervention group; 34.5%) reported in the literature [15].

### 2.2. Design

This study was designed as a prospective RCT, comprising two parallel arms (oral care and control groups). The RCT was conducted across four rehabilitation units of a medical centre in Taiwan. The first author used Random Allocation Software 2.0 to randomly assign patients to one of the two groups.

The oral care group received the intervention over the course of 3 weeks. Assessment of the outcome variables was conducted one day before the start of the swallowing therapy (T1), 2 weeks after the swallowing treatment (T2), and 3 weeks after the swallowing treatment (T3) by a research assistant blinded to the group allocation. The participants in the control group completed the outcome measures in the same timeframe as those in the oral care group. Approval for this trial was obtained from the Institutional Review Board of the hospital (IRB no: 201700143B0). This study was registered with ClinicalTrials.gov (identifier: NCT03219346).

### 2.3. Participants

Sixty-six patients (23 female, 43 male) with dysphagia following a first-time stroke, in four rehabilitation units in northern Taiwan, who received swallowing treatment, were invited to participate in this study. The patients also had to be able to communicate in Chinese (Mandarin or Taiwanese), comply with the instructions, and be willing to participate in this study.

**Figure 1:**
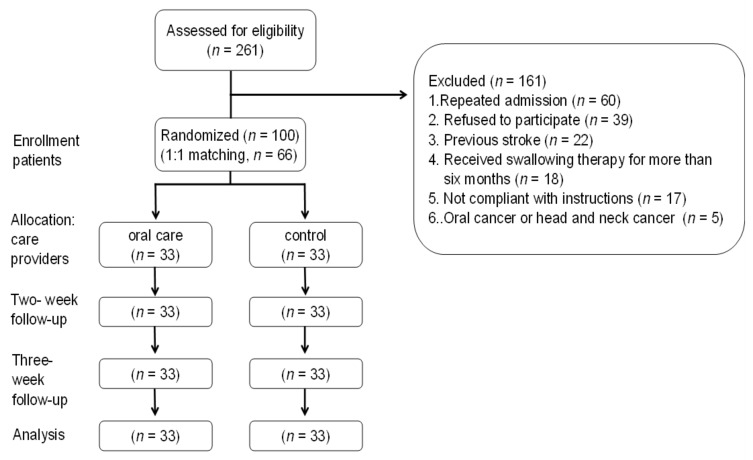
Consort flow diagram for oral care randomized controlled trial (RCT).

The exclusion criteria were a history of dysphagia because of oral cancer or head and neck cancer [30] and having already received more than 6 months of swallowing treatment (Figure 1).

### 2.4. Intervention

#### 2.4.1. The Control Group

Patients in the control group received the usual oral care provided in the unit (e.g., tooth brushing or sponge stick cleaning) twice a day (morning and evening) and were provided an instructional manual to promote eating (including information such as food choice and safe eating tips) [31].

#### 2.4.2. The Oral Care Group

Besides the usual oral care and manual provided to the control group, the patients in this group received oral health care 30 minutes before the swallowing training three times a week for 3 weeks.

The primary author instructed the caregiver on how to perform the oral health procedure until the caregiver was confident in performing the procedure independently, taking 10–15 minutes each time. Before providing oral health care, the caregiver had to prepare the necessary oral health tools (such as water, toothbrush, dental floss, and interdental brush) and suction equipment (including saliva pipette) and help the patient sit in an upright position. First, the patient’s sputum in the oral cavity was assessed. A suction was used to clear the saliva when necessary [20]. Next, an oral cleaning tool (dental floss and/or interdental brush) was used [15], and the patient’s teeth were brushed using the Bass method. Finally, a fluoride toothpaste (fluoride >1,000 ppm, <0.5 cm used to prevent caries) was used to coat all teeth [32].

### 2.5. Variables Measured/Calculated

#### 2.5.1. Oral Health Assessment Tool (OHAT)

A modification of the Simple Oral Health Status Test Scale developed by [33] resulted in the creation of the Oral Health Assessment Tool (OHAT) scale, which clinical nurses can use to assess oral health during the daily function assessment of elderly adults. The OHAT assesses eight items: the condition of the lips, tongue, gums and mucous membranes, saliva, residual teeth, and dentures, as well as the presence of toothache and the degree of oral cleanliness. The score for each item ranges from 0 to 2 points, and the total score ranges from 0 to 16 points. Higher scores denote worse oral health. The OHAT Cronbach’s α was reported to be 0.60 and the simultaneous validity (Kappa value: 3.162–4.337) indicated a significant correlation (*p* < 0.05) [34].

#### 2.5.2. Functional Oral Intake Scale (FOIS)

The FOIS is a tool commonly used to measure oropharyngeal dysphagia [35]. The FOIS involves seven levels. Levels 1 through 3 relate to varying degrees of non-oral feeding, while levels 4 through 7 relate to varying degrees of oral feeding without non-oral supplementation. Operational definition data were normalised (the FOIS has been published in Chinese) and the scale has good reliability and validity [36,37].

#### 2.5.3. Mini-Nutritional Assessment-Short Form (MNA-SF)

The Mini-Nutritional Assessment-Short Form (MNA-SF) questionnaire was used to record the patient’s diet, weight, mobility, mental stress, neuropsychiatric disorders, and upper arm circumference over the past 3 months [38]. It has good reliability and validity [39].

A score of 13 points indicates normal nutritional status, 12 points indicates low risk of malnutrition, 8–11 points indicates potential risk of malnutrition, and a score of 7 points or lower indicates high risk of malnutrition [38].

#### 2.5.4. Rate of Nasogastric Tube Removal

The investigators used on-site observation to confirm whether the nasogastric tube was in situ. If not present, the removal date was recorded, e.g., in the second week of the swallowing treatment (T2) or the third week of the swallowing treatment (T3). In the rehabilitation ward, the criteria for removal of a nasogastric tube include: (1) The patient is able to receive oral medication, (2) the patient can drink water up to 1500 ml per day, and (3) the patient is able to consume soft food without coughing. In addition to these three conditions, confirmation from the physician and the speech therapist are needed before removing the nasogastric tube.

### 2.6. Statistical Analysis

To compare the oral care group and the control group, we performed propensity score matching. The propensity score was the predicted probability of being in the oral care group given the values of the covariates. These covariates included all the variables listed in Table 1. Statistical analyses were conducted using MatchIt 3.5.2 (Comprehensive R Archive Network, Princeton, NJ, USA) and SPSS Version 22.0 (SPSS Inc., Armonk, NY, USA). The baseline data for the two groups were compared using the chi-square test for categorical variables and the t-test for differences between groups. The baseline data in this study were homogeneous. Inferential analysis of repeated measures was conducted using a generalised estimating equation (GEE) to examine the effectiveness of the interventions in improving oral intake status (OHAT, FOIS, MNA-SF) relative to the control group. We also used the chi-square test to compare the nasogastric tube removal rates between groups.

## 3. Results

### 3.1. Baseline Characteristics of the Two Groups 

A total of 66 patients with stroke and dysphagia were recruited. There were no significant differences between the two groups (*p* > 0.05) in the baseline characteristics listed in Table 1. Regarding the oral intake status (FOIS, OHAT, MNA-SF) at baseline, there was no significant difference between the two groups (*p* > 0.05) (Table 2).

### 3.2. Impact of the Experimental Programme on the Outcomes

#### 3.2.1. OHAT

**Figure 2:**
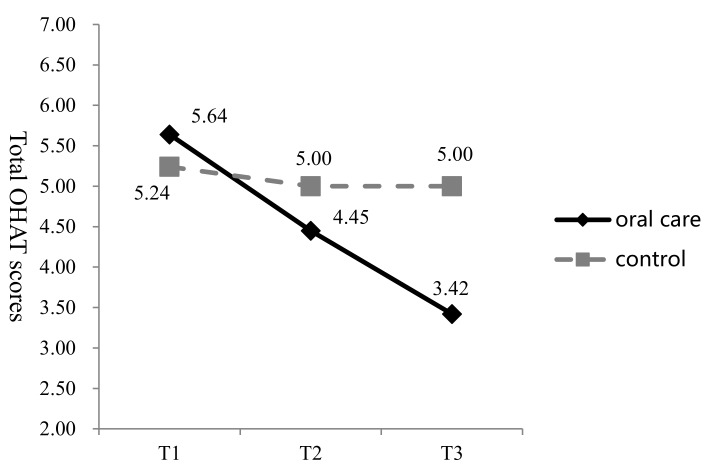
Change in the Oral Health Assessment Tool (OHAT) scores in the two groups at three time points (T1–T3).

At T1, T2, and T3, the mean OHAT values in the oral care group were 5.64 (standard deviation (SD) = 2.54), 4.45 (SD = 2.29) and 3.42 (SD = 1.89), respectively. At the same time points, the average scores in the control group were 5.24 (SD = 1.77), 5.00 (SD = 1.95), and 5.00 (SD = 2.14), respectively (Figure 2). In the oral care group, the mean OHAT decreased from T1 to T3 by 2.22 points compared to a decrease of 0.24 points in the control group. GEE analysis showed a statistically significant improvement in the OHAT score in the oral care group at T3 (Table 3). The interaction effect also reached significance (95% CI = −2.69 to −1.25, Wald **χ^2^** = 29.02, *p* < 0.001), indicating that the change in OHAT over time in the oral care group was greater than that in the control group.

#### 3.2.2. FOIS

**Figure 3:**
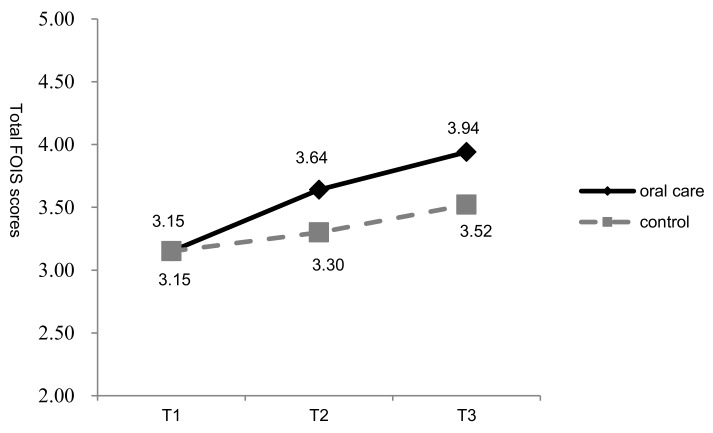
Change in the mean Functional Oral Intake Scale (FOIS) score in the two groups at three time points (T1–T3).

At T1, T2, and T3, the mean FOIS values of the oral care group were 3.15 (SD = 2.06), 3.64 (SD = 2.29), and 3.94 (SD = 2.38), respectively. At the same time points, the mean FOIS scores in the control group were 3.15 (SD = 1.79), 3.30 (SD = 1.86), and 3.52 (SD = 1.92), respectively (Figure 3). In the oral care group, the mean FOIS score at T3 was 0.79 points higher than that at T1, compared to a respective increase of 0.37 in the control group. Despite higher FOIS scores in the oral care group than in the control group, GEE analysis indicated no statistical significance in the improvement in FOIS score in the oral care group at T3. The interaction effect did not reach significance (95% CI =−0.16 to.89, Wald **χ^2^** = 1.86, *p* > 0.05) (Table 3).

#### 3.2.3. MNA-SF

**Figure 4:**
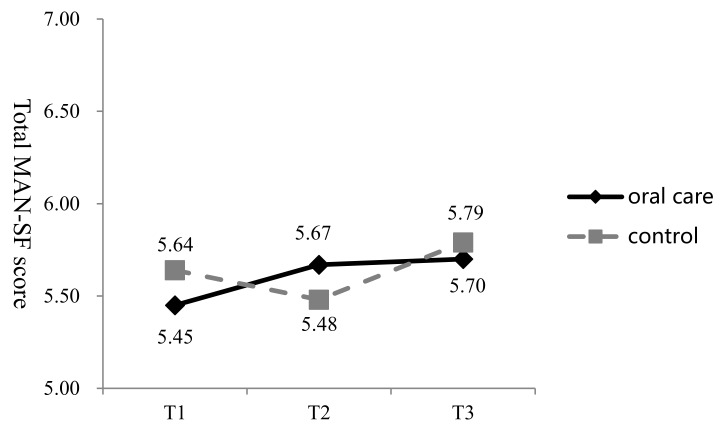
Change in the Mini-Nutritional Assessment-Short Form (MNA-SF) scores in the two groups at three time points (T1–T3).

The mean MNA-SF score in the oral care group was 5.45 (SD = 2.48), 5.67 (SD = 2.59), and 5.70 (SD = 2.60) at T1, T2, and T3, respectively. In the control group, the respective scores were 5.64 (SD = 2.03), 5.48 (SD = 2.17), and 5.79 (SD = 2.37) (Figure 4). In the oral care group, the mean MNA-SF at T3 was 0.25 points higher than that at T1, compared to a respective increase of 0.15 in the control group. The GEE analysis showed a statistically non-significant improvement in the MNA-SF in the oral care group at T3 (interaction effect, 95% CI = −0.35 to 0.53, Wald **χ^2^** = 0.17, *p* > 0.05) (Table 3). GEE analysis indicated that the observed longitudinal difference in scores between the two groups was not statistically significant.

#### 3.2.4. Nasogastric Tube Removal

**Figure 5:**
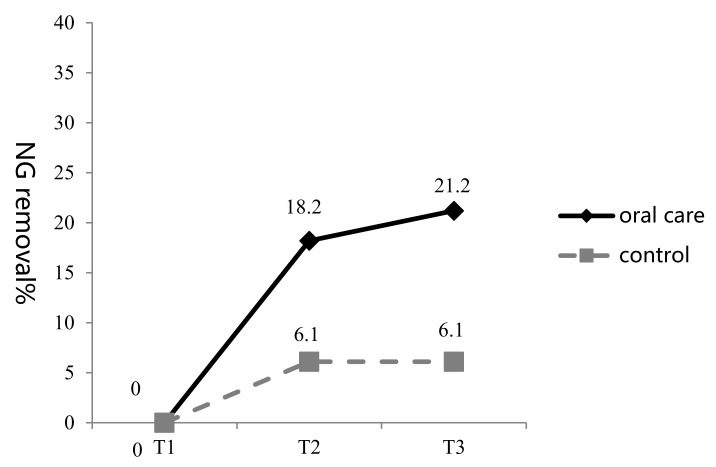
Changes in the percentage of nasogastric tubes removed in the two groups at three time points (T1–T3).

The nasogastric tube was in situ in both groups at T1. At T2, the nasogastric tube had been removed in two (6.1%) and six (18.2%) patients in the control and oral care groups, respectively. At T3, there were two (6.1%) and seven (21.2%) nasogastric tube removals in the two groups, respectively (Figure 5).

Results of the chi-square test showed that the difference between the two groups in the number of nasogastric-tube removals at T3 was not statistically significant (*p* > 0.05), although more patients in the oral care group than in the control group had their nasogastric tubes removed (Table 4). 

Therefore, the results showed that the null hypothesis could be partially rejected. 

## 4. Discussion

This study examined the efficacy of an oral health programme before swallowing treatment to improve oral health, oral intake, and nutrition among patients with stroke with dysphagia in Taiwan. Based on the results, the null hypothesis was partially rejected. Following 3 weeks of the oral health programme, significant improvements were noted in the oral health of the patients in the oral care group relative to that of the control group; however, there were no significant differences in the FOIS and MNA-SF scores or the rate of nasogastric tube removals (*p* > 0.05). With regards to the FOIS, the oral care group had higher mean scores than the control group (3.94 vs. 3.52, respectively), but the difference was not statistically significant (*p* > 0.05). This lack of significance may be because the intervention lasted only 3 weeks, making it difficult to assess the effect of the intervention on the FOIS scores, nasogastric tube removal, and nutritional assessments in such a short period. We are hopeful that future studies with longer durations will address the needs of patients with stroke and a history of dysphagia for 3 months or longer, to enhance the effectiveness of the swallowing therapy as well as the application of the programme in long-term care.

Improvements in oral health, shown by the significant decline in the oral health assessment score (OHAT) after receiving the oral health intervention, suggest that oral hygiene may improve the oral health of patients with stroke and dysphagia. This evidence-based oral health programme can diminish the barriers to success in swallowing therapy, residual sputum in the mouth, and poor oral hygiene [13]. Regarding the OHAT, we found that 21 of the 66 patients with stroke (31.8%) had symptoms of dry mouth, three (4.5%) had toothache, and 15 (22.7%) had active dentures, but 11 (73.3%) of these 15 patients did not wear dentures because they neglected to bring them to the hospital or simply did not wear them. The results of this study are consistent with those of another study [40] that found that elderly patients with dysphagia are prone to chewing disorders. Caregivers should, therefore, assist patients in managing oral pain and reiterate the importance of carrying removable prostheses when visiting the hospital.

Regarding the oral food intake status as assessed by the FOIS, at T3, 10 subjects (three subjects ≥65 years old, seven subjects <65 years old) consumed all food orally without limitations. We also found that 10 subjects (two in the control group vs. eight in the oral care group) showed improved oral food intake at T3. Although statistically insignificant, the oral care group had higher FOIS scores than did the control group (3.94 vs. 3.52). These results are similar to those of previously published studies [15]. The oral health care programme intervention improved oral food intake in patients. However, due to the influence of ageing, oral food intake in elderly patients was less efficient than that in younger subjects. This difference makes the issue of elderly nutrition worthy of our continued attention.

There were no significant changes in the nutritional status in either group. The nutritional status was assessed using the MNA-SF; at T1, most subjects (40, 60.6%) had moderate anorexia due to dysphagia. Half of the subjects (33, 50.0%) had weight loss of 1–2 kg. At T1, 42 subjects (28 subjects ≥65 years old and 14 subjects <65 years old) were at high risk of malnutrition (63.6%). The results of this study are similar to those of previous studies [3,41]. Malnutrition is associated with dysphagia. Elderly patients (57.8%) account for the majority of subjects at high risk of malnutrition [3]. Because most elderly patients have poor appetite, and oral food intake function is poor, weight loss occurs within 3 months of stroke, placing elderly people at high risk of malnutrition. In addition, six subjects were found to have aphasia during the assessment phase. It has been reported that dysphagia and aphasia occur in isolation or concomitantly in two-thirds of all patients with first-ever acute ischaemic stroke [42]. It has been previously highlighted that, in subjects with post-stroke dysphagia, the decline in language function forces them to express their emotions through certain behaviours, which include refusing food and not obeying the instructions of their primary caregivers [43]. In this study, the interactions and communication with primary caregivers were consistent with those reported in the literature.

In this study, we found that nine subjects (two in the control group vs. seven in the oral care group) had their nasogastric tubes removed at T3. An increased rate of nasogastric-tube removal was noted in the oral care group (21.2% vs. 6.1%, respectively). However, this difference was statistically nonsignificant. This result is similar to findings by [15], who reported that oral health care can improve the outcomes associated with oral food intake function. 

### 4.1. Limitations

There are several limitations to this study. First, the National Health Insurance policy limits patients with stroke to 4 weeks of hospitalisation at a time for swallowing training. For this reason, the intervention in this study was limited to 3 weeks. However, changes in one of our outcome variables, MNA-SF, could require up to 3 months to appear [38]. Secondly, the sample size was relatively small. In our four study units, there were, per month, approximately ten patients with first-time stroke receiving swallowing therapy. Furthermore, patients with repeat hospitalisations were excluded. Therefore, the sample size was overall relatively small, even though we recruited participants for 12 months.

### 4.2. Future Directions

Our research conclusions necessitate further support by a larger study with a longitudinal design based in diverse hospitals. Furthermore, other variables should be measured, such as the relationship between systemic diseases and oral health-related quality of life (OHRQoL).

## 5. Conclusions

This 3 week oral health programme, if provided before swallowing treatment, could improve oral health outcomes and help maintain oral intake among patients who have had a stroke with dysphagia. Therefore, clinicians can apply this oral health programme as a ward routine during the rehabilitation of patients with a dysphagic stroke who need to undergo swallowing training, to promote oral health and functional oral intake.

## Figures and Tables

**Table 1:** Baseline characteristics of the oral care and control groups.

Variable	Total (*n* = 66)		Oral Care Group (*n* = 33)		Control Group (*n* = 33)		χ^2^	*p*
	Frequency (%)		Frequency (%)		Frequency (%)			
Sex							1.67	0.196
Male	43	65.2	19	57.6	24	72.7		
Female	23	34.8	14	42.4	9	27.3		
Age							0.000	1.000
≥65 years	36	54.5	18	54.5	18	54.5		
<65 years	30	45.5	15	45.5	15	45.5		
Stroke type							0.061	0.805
Infarction	35	53.0	18	54.5	17	51.5		
Haemorrhagic	31	47.0	15	45.5	16	48.5		
Unilateral location							2.63	0.295 a
Right	34	51.5	20	60.6	14	42.4		
Left	28	42.4	12	36.4	16	48.5		
Non-unilateral	4	9.1	1	3.0	3	9.1		
Interval of OHP ^b^ (months)	0.5 (0.5–2)		0.5 (0.5–2)		0.5 (0.5–2)		−0.373	0.709
Swallowing severity							0.000	1.000
Mild	24	36.4	12	36.4	12	36.4		
Moderate	28	42.4	14	42.4	14	42.4		
Severe	14	21.2	7	21.2	7	21.2		
Active denture							2.16	0.142
No	51	77.3	23	69.7	28	84.8		
Yes	15	22.7	10	30.3	5	15.2		
Actively wearing dentures (*n* = 15)							0.68	0.560 ^a^
No	11	73.3	8	80.0	3	60.0		
Yes	4	26.7	2	20.0	2	40.0		

Interval of OHP (months): time interval from stroke onset to the date of the oral health programme (OHP). ^a^ Fisher’s exact test, ^b^ the Mann–Whitney test.

**Table 2:** Baseline Oral Health Assessment Tool (OHAT), Functional Oral Intake Scale (FOIS), and Mini-Nutritional Assessment-Short Form (MNA-SF) scores between the oral care and control groups.

Outcome	Group	Pre-Oral Care		*t*	*p*
		Mean (SD)	*n*		
OHAT	oral care	5.64 (2.54)	33	0.732	0.467
	control	5.24 (1.77)	33		
FOIS	oral care	3.15 (2.06)	33	0.000	1.000
	control	3.15 (1.79)	33		
MAN-SF	oral care	5.45 (2.48)	33	−0.326	0.745
	control	5.64 (2.03)	33		

**Table 3:** Results of the Generalised Estimating Equation analysis on the effectiveness of the oral health programme on the outcome variables.

Variables	Regression Coefficients	Standard Error	95% CI of *B*	Wald χ^2^	*p*
**OHAT**	5.24	0.30	4.65–5.84	299.11	0.000
Group (OC) ^†^	0.39	0.53	−0.64–1.43	0.55	0.457
Time 3 ^‡^	−0.24	0.25	−0.73–0.24	0.97	0.325
Time 2 ^‡^	−0.24	0.21	−0.66–0.18	1.28	0.258
Interaction					
Group (OC) × Time 3 ^§^	−1.97	0.36	−2.69-(−1.25)	29.02	0.000
Group (OC) × Time 2 ^§^	−0.94	0.30	−1.54-(−0.34)	9.52	0.002
**FOIS**	2.74	0.27	2.21–3.27	101.92	0.000
Group (OC)^†^	−0.27	0.43	−1.11–0.56	0. 41	0.523
Time 3^‡^	0.17	0.16	−0.14–0.49	1.22	0.270
Time 2^‡^	−0.05	0.10	0.10–0.15	0.24	0.625
Interaction					
Group (OC) × Time 3 ^§^	0.37	0.27	−0.16–0.89	1.86	0.172
Group (OC) × Time 2 ^§^	0.35	0.19	−0.02–0.71	3.36	0.067
**MNA-SF**	5.64	0.35	4.96–6.32	262.82	0.000
Group (OC)^†^	−0.18	0.55	−1.26–0.89	0.11	0.740
Time 3^‡^	0.15	0.18	−0.20–0.50	0.73	0.393
Time 2^‡^	−0.15	0.16	−0.46–0.15	0.95	0.329
Interaction					
Group (OC) × Time 3 ^§^	0.09	0.22	−0.35–0.53	0.17	0.684
Group (OC) × Time 2 ^§^	0.36	0.20	−0.03–0.76	3.29	0.070

SE, standard error of the mean; CI, confidence interval; OC, oral care; OHAT, oral health assessment tool; FOIS, functional oral intake scale; MNA-SF, Mini-Nutritional Assessment-Short Form. ^†^ Reference group, control group. ^‡^ Reference group, time (1st). ^§^ Reference group, group (CON) × Time (1st).

**Table 4:** Analysis of the effects of the oral health programme on nasogastric tube removal (*n* = 66).

Variable	Control (*n* = 33) *n*	%	Oral Care (*n* = 33) *n*	%	χ^2^	*p*
NG removal (T2)	2	6.1	6	18.2	2.28	0.131
NG removal (T3)	2	6.1	7	21.2	3.22	0.073

NG, nasogastric tube; T2, time 2; T3, time 3.
